# PATHWAYS TO RESILIENCE: TRANSFORMING TRAUMA THROUGH THE POWER OF NETWORKS

**DOI:** 10.1093/geroni/igz038.2958

**Published:** 2019-11-08

**Authors:** Connie Corley, Maureen Feldman, Scott Kaiser

**Affiliations:** 1 Fielding Graduate University, Santa Barbara, California, United States; 2 Los Angeles Pierce Community College, Woodland Hills, California, United States; 3 University of Southern California, Los Angeles, California, United States

## Abstract

Resilience has been examined in various age groups, initially focused on vulnerable children and more recently in enhancement of resilience in various age groups and in response to trauma. Based on studies of resilience in Holocaust survivors and intergenerational engagement to promote resilience in former gang members and isolated older adults, Corley’s 3E model of Experience, Expression and Engagement is discussed in terms of multiple studies and the implications for forming and strengthening networks in communities at risk. This includes initiating creative coalition-building endeavors to address loneliness in residential settings for older adults evolving from a project funded to the Motion Picture and Television Fund in Los Angeles from the AARP Foundation.

